# Finasteride and Male Breast Cancer: Does the MHRA Report Show a Link?

**DOI:** 10.4103/0974-2077.69022

**Published:** 2010

**Authors:** Niraj K Shenoy, Sangolli M Prabhakar

**Affiliations:** *#301-B, Ranka Colony, Bilekahalli, Bannerghatta Road, Bangalore - 560 076, India*; 1 *11^th^ Cross, Gayathrinagar HBCS Layout, Basaveshwaranagar, Bangalore - 560 079, India*

**Keywords:** Finasteride, breast cancer, male

## Abstract

Finasteride is an important drug for the management of androgenetic alopecia. However, there are concerns about the possible side effects of the drug such as impotence. Recently stray reports have appeared about the occurrence of male breast carcinoma in patients who received the drug. These have been looked in to by Medicines and Health care products Regulatory Agency (MHRA). This article summarizes the MHRA report.

## INTRODUCTION

Finasteride is a competitive and specific inhibitor of type II 5α-reductase, an enzyme involved in the conversion of testosterone to dihydrotestosterone (DHT), which is the main androgen involved in the pathogenesis of benign prostatic hyperplasia (BPH) and androgenetic alopecia. Five milligrams finasteride daily is used as a treatment for BPH and 1 mg Finasteride daily is used in the treatment of androgenetic alopecia. Inhibition of type II 5a-reductase with finasteride results in significant and rapid decrease in serum and tissue DHT concentrations, with significant suppression of DHT reached within 24 hours of dosing. Finasteride inhibits DHT-mediated effects on target tissues without lowering serum testosterone levels or affecting testosterone-mediated effects in tissues.[1]

There have been several questions raised about the safety profile of finasteride. Possible side effects such as decreased libido and other sexual side effects, like gynecomastia, and possible link to prostatic cancer have been the topics of debate.[2–5] Since finasteride is an important drug in the management of androgenetic alopecia and BPH, this issue has received a lot of publicity, particularly over the Internet. While several studies have established its safety, there is generally a great hesitation to accept the drug among patients, particularly because the drug is not completely curative, and hence, needs to be taken for several years.

In addition to these previously reported side effects, recently a possible and rather disturbing effect is also observed. There has been an increase in the number of case reports of male breast cancer associated with the use of finasteride as pointed out in a recent report[6] of Medicines and Health care products Regulatory Agency (MHRA). MHRA is the government agency responsible for the regulation medicines and medical devices in the United Kingdom (UK). They regularly review safety of all medicines in UK and inform health care personnel and public of the latest updates. In public assessment reports, they discuss evidence-based concerns associated with a particular drug or its class. Present report presents a summary of the MHRA report on the possible risk of carcinoma of breast in males with long term usage of finasteride.

## BACKGROUND INFORMATION ABOUT CARCINOMA BREAST IN MEN

Male breast cancer is a rare disease with an incidence in general population of 1/100000 man- years which is 100 times less in comparison to the incidence observed in finasteride-treated population.[7] The consequence of this rarity is that male breast cancer has been understudied in comparison to female breast cancer, and the existing small sample size limits the epidemiological methodology available to study the disease. In men, its behaviour is similar to breast carcinoma in postmenopausal women. Age of onset is between 52–71 years in females and 71 years in males.[8] The clinical manifestations of male breast cancer include breast mass (seen in 75% of patients), nipple retraction (9%), nipple discharge (6%), skin/nipple ulceration (6%) and Paget’s disease of the nipple (1%).[9] The survival rate for men with breast cancer is lower than the rate of survival for women with the same disease because of their increased age at diagnosis and more advanced stage of the disease at presentation. Carcinoma of breast is more often than not an oestrogen receptor sensitive tumour. The source of oestrogen in males is conversion of testosterone to oestrogen and androstenedione to oestrone in fat cells.[10] Survival rate is less in male patients due to the advanced age of patients, advanced stage of disease at the time of detection and lack of awareness in men.[11] However, there is no significant difference in survival rates in men in comparison with female patients if age-matched and stage-matched incidence is compared.[12] Nearly 20% of male patients have a family history of carcinoma of breast among female relatives.[13] Risk factors for male breast carcinoma include BRCA1 and BRCA2 mutations, Klinefelter’s syndrome, altered testosterone and oestrogen balance, testicular disorders, obesity, carcinoma of prostate and its treatment. Contradictory evidence has been reported in literature about gynecomastia as a risk factor for breast cancer in males with evidence both for[14] and against it.[15]

## REVIEW OF DATA ABOUT MALE BREAST CARCINOMA OCCURRING IN PATIENTS RECEIVING FINASTERIDE

As of November 2009, there have been 50 worldwide case reports of male breast cancer in BPH patients aged between 54 and 88 years (mean age of 71 years), who received 5 mg finasteride. The time to onset could be estimated in 35 of the reports; a mean time to onset being approximately 44.4 months from the commencement of treatment. The median time to onset was 36 months (range: 5 weeks–11 years). Twenty-seven cases occurred after finasteride treatment for a minimum of 1 year. Three cases have been reported with the use of Propecia® 1 mg for androgenetic alopecia. Of the 3 cases of male breast cancer reported with Propecia® 1 mg, inadequate information and the relatively short times to onset in these cases makes the causal association between male breast cancer and finasteride unlikely.[6]

### Pre-clinical data

In rats, high dose of finasteride for a long period did not result in malignancy.[16] However, clinical data have emerged on this subject, which are reviewed below.

### Clinical data

#### Data from UK

Five cases of carcinoma of breast were retrieved from the Adverse Drug Reaction (ADR) profile involving finasteride for treatment of BPH.[17] Gynecomastia was the most common ADR in reproductive and breast disorders system organ class (SOC). A total of 75 cases of carcinoma of breast were recorded (69 with 5 mg/d, 4 cases with 1 mg/d, 2 cases the dosage was unknown).

#### Data from world literature

Worldwide search yielded 50 cases in BPH patients including 44 cases with medically proven reports. Time to onset could be estimated in 35 cases, with the mean being 44.4 months from the commencement of treatment (range: 5 weeks–11 years). Out of the 35 cases, 27 developed malignancy more than one year after treatment initiation and 8 developed carcinoma within 1 year after initiation of therapy. In 7 patients, finasteride was discontinued (7–24 months) before the onset of cancer. Eight patients continued finasteride even after the detection of malignancy. In 3 cases, finasteride was administered for only a few months before the appearance of malignancy, and hence, the drug could not be regarded to have been causative. Under reporting is the major problem in ADR scheme.[18]

Also, 4 cases of carcinoma of breast have been reported in female patients within 1 year of finasteride administration. It is possible that short duration of finasteride therapy may be sufficient to induce carcinoma of breast in women because of the presence of endogenous oestrogen.

#### Proscar long term efficacy and safety study

Proscar long term efficacy and safety study (PLESS), 3040 patients were followed up for a period of 4 years.[19] The patients were randomised in approximately equal proportions to receive either 5 mg finasteride or placebo for up to 4 years In this study, there were no cases of male breast cancer reported in finasteride-treated subjects, and 2 cases were reported in placebo-treated subjects.

#### Medical therapy of prostatic symptoms

In this study,[20] 3047 patients were randomised to a double-blind, multi-centre, placebo-controlled clinical trial for 4-6 years. The 4 different patient groups were administered different drugs: placebo; 8 mg doxazosin; 5 mg finasteride and a combination of 8 mg doxazosin and 5 mg finasteride. Three cases of breast cancer occurred in the finasteride-treated group and 1 case of breast cancer occurred in the combination group. No predisposing factors were identified. Duration of treatment ranged from 1.8 years to 5 years. The occurrence of 4 cases of breast cancer in 3047 patients was considered high considering the incidence in the general population of 1 case in 100,000 man-years. There was temporal relationship in all the 4 cases and it cannot be stated to have occurred by chance, as suggested by some authors. Treatment with finasteride appeared to confer 200-fold risk for breast cancer in comparison to patients not receiving the drug.

#### Prostate cancer prevention trial (PCPT)

In the PCPT study,[3] 18882 men aged 55 years or older with a normal digital rectal examination and a prostate-specific antigen (PSA) level of 3 ng/mL or lower were randomized to treatment with 5 mg/day finasteride (*n* = 9423) or placebo (*n* = 9459) for 7 years. Prostate biopsy was recommended if the annual PSA level, adjusted for the effect of finasteride, exceeded 4 ng/mL or if the result of a digital rectal examination was abnormal. The primary endpoint was the prevalence of prostate cancer during the 7 years of the study. One case of breast cancer was reported as an adverse experience in each treatment group during the study. The temporal relationship of 7 years makes a causal association in the finasteride case possible. However, a similar time to onset of 6 years was observed in the placebo case. In this very large long-term study, an increased incidence of breast cancer in the finasteride group compared to placebo was not observed.

#### Prescription event monitoring

Was conducted by Drug Safety Research Unit (DSRU).[4] In a non-intervention observation cohort study, a total of 14772 patients were under observation of General Practitioners from 1992–1994. Of the patients, all but 5 were male (2 were female and the gender was unknown for 3 patients), and the average age was (mean [standard deviation]) 69 (9·2) years. The indication for use in 83% of the cases was prostatism and related conditions, and was unspecified in 15%, although 1 female was treated with finasteride for hirsutism. During this period, 95 events with finasteride use were reported as ADRs in 75 patients. The most frequent ADR reported was impotence, with impotence or ejaculatory failure occurring in 2.1% of the cohort. Gynecomastia and related conditions occurred in 0·4% of the cohort; 17 patients experienced other unspecified breast disorders and mastalgia occurred in 4 patients. A total of 33 events concerning malignancies were reported in patients, of these, 2 were reported as breast carcinoma. For one of the events, the time to onset from commencement of finasteride treatment was recorded as 5 months, the other was unknown. Four events of non-malignant breast tumour were also recorded but no further information is known about these cases.

The PEM study concludes overall that that finasteride is acceptably safe when used in accordance with the current prescribing information. However, it is not possible from this study to evaluate the cases of breast cancer and their causal relationship with finasteride, as enough data is not available regarding the 2 events of breast carcinoma and 4 events of non-malignant breast tumour. Furthermore, the short period of observation limits the possible number of cases which could be identified within this period.

#### Other reports

Other reports have revealed confusing data. Food and Drug Administration (FDA) reported 13 cases who had received finasteride for BPH between 1992 and 2003.[21] Average age of the patients was 66 years. Duration of the treatment was 21 months. Among the 13, 4 patients had gynecomastia. Carter[22] reported 2 cases, 1 on finasteride for 35 days (age-59 years), and the other on finasteride (age-63 years) for 21 months.

Surprisingly, 3 cases of cancer of breast have been reported in patients on selective serotonin reuptake inhibitors (SSRI).[23] Ekman did not find any patient with breast cancer in his study of patients on finasteride for BPH.[5] Gynecomastia was not considered a risk factor for malignancy in finasteride treated patients as its incidence is only 9%[24] as compared to 50% incidence in general population.

## POTENTIAL BIOLOGICAL MECHANISMS

Risk factors for breast carcinomas stated earlier include age, Klinefelter’s syndrome, genetic mutations, family history of breast cancer, radiation exposure, alcohol cirrhosis, chronic liver disease leading on to hyperoestrogenism, testicular diseases like cryptorchidism, orchitis, orchiectomy, testicular injury, obesity, working in hot environments, exposure to gasoline fumes, benign breast conditions like nipple discharge, breast cyst and Jewish ancestory.[25]

The mechanism by which finasteride could cause male breast cancer is thought to be via altering hormone levels. As stated earlier, finasteride leads to altered oestrogen/testosterone balance. Role of oestrogen in breast carcinoma is considered important. Testosterone is converted to oestradiol and androstenedione is converted to oestrone in fat cells. Increased oestrogen/testosterone ratio in a patient may lead to malignant transformation.[26] Oestrogen-blocking drug like tamoxifen and aromatase inhibitor anastrozole prove this point.[27] Either the slight increase in oestrogen or the reduction in the ratio of potency of androgens to that of oestrogens (due to reduced DHT levels) or both could be the responsible mechanism/s in finasteride-associated male breast cancer.

Oestrogen is genotoxic, mutagenic and has transforming and carcinogenic potential.[28] It increases telomerase activity which causes cell division, hence may cause neoplasia. Increased concentration of testosterone leads to its conversion to oestradiol resulting in increased levels of oestrogen as has been established in finasteride (1 mg/d) study by Vaughen *et al*.[29]

## CONCLUSION

The exhaustive and meticulous analysis of data presented in MHRA report need to be considered seriously and the matter needs to be investigated further. Whether the reported link needs to be informed during the patient’s counselling is a matter under consideration of the drug authorities. Any symptom suggestive of breast pathology (nipple discharge, pain or swelling in the breast) in a patient on finasteride needs to be seriously examined and investigated thoroughly. MHRA report is available on the internet, which may be read by prospective patients and hence treating physicians need to be aware of the report so that proper counseling can be provided. Topical finasteride, in better pharmulations, to ensure proper absorption, is a possible alternative for management androgenetic alopecia. This also underscores the need for greater research in to the pharmacological treatment of androgenetic alopecia.
